# Transferable G/Au Film for Constructing a Variety of SERS Substrates

**DOI:** 10.3390/nano14070566

**Published:** 2024-03-25

**Authors:** Xinyu Zhang, Xin Cai, Naiqiang Yin, Yingying Wang, Yang Jiao, Chundong Liu

**Affiliations:** 1 School of Physics and Electronic Engineering, Qilu Normal University, Jinan 250200, China; 2 School of Physics and Electronics, Shandong Normal University, Jinan 250014, China

**Keywords:** surface-enhanced Raman scattering, graphene, electromagnetic enhancement mechanism, chemical enhancement mechanism

## Abstract

Surface-enhanced Raman scattering (SERS), as one of the most powerful analytical methods, undertakes important inspection tasks in various fields. Generally, the performance of an SERS-active substrate relies heavily on its structure, which makes it difficult to integrate multiple-functional detectability on the same substrate. To address this problem, here we designed and constructed a film of graphene/Au nanoparticles (G/Au film) through a simple method, which can be conveniently transferred to different substrates to form various composite SERS substrates subsequently. By means of the combination of the electromagnetic enhancement mechanism (EM) and the chemical enhancement mechanism (CM) of this structure, the film realized good SERS performance experimentally, with the enhancement factor (EF) approaching ca. 1.40 × 10^5^. In addition, the G/Au film had high mechanical strength and had large specific surface area and good biocompatibility that is beneficial for Raman detection. By further transferring the film to an Ag/Si composite substrate and PDMS flexible film, it showed enhanced sensitivity and in situ detectability, respectively, indicating high compatibility and promising prospect in Raman detection.

## 1. Introduction

SERS has been used in various fields such as biomedicine, chemical analysis, and environmental analysis due to its powerful qualitative analysis capabilities [1,2,3,4,5,6]. Two mechanisms are widely recognized to explain the enhancement effect of SERS: the EM caused by the local plasmon effect of the noble metal nanostructure and the CM mainly caused by the charge transfer of two-dimensional materials [7,8,9]. At present, it has been confirmed through a large number of reports that EM is the main contribution provider of the SERS effect, and its enhancement factor can reach 10^14^, while CM can provide an additional enhancement factor to further improve the detection sensitivity. Therefore, preparing nanocomposite structures of noble metals and two-dimensional materials that can realize both EM and CM on the same SERS substrate is currently a popular method [10,11]. In addition, these nanocomposites have additional advantages compared to pure metal nanostructures or two-dimensional materials. For example, two-dimensional materials can be used in spacer layers of metal structures to construct suitable ‘hot spots’ with tiny nano-gaps. Nanocomposites can increase the specific surface area of the SERS substrate and provide more binding sites to analyte molecules, and the functional groups on the surface of the two-dimensional materials can adsorb biomolecules [12,13]. Therefore, it is crucial to select suitable two-dimensional materials and metals for constructing metal nanocomposite SERS substrates. However, there are still a lot of obstacles in the actual detection of SERS technology, among which the most critical problem is that different bases of different properties are required for different detection needs. For general substrates, in the detection process, it is difficult to ensure that the measured object can effectively enter the hot spot range, which directly affects the stability and reliability of the detection. How to solve this problem has been a research focus of researchers in this field in recent years, and the optimization of SERS-related methods and materials is the most important research. Some strategies, such as constructing gold nanostars [14] and self-assembling gold nanospheres [15], have been proposed.

Graphene has been favored in various fields due to its excellent mechanical, electrical, and optical properties; moreover, it is often used as an ideal material for SERS substrates due to its excellent characteristics, such as the large specific surface area, significant CM capability, and effective background fluorescence suppression [16]. At present, graphene and noble metals with strong enhancement effects, such as Au, Ag, and Cu, are usually prepared into hybrid structures to build the SERS substrate [17,18]. Among these noble metals that are chosen frequently, Au has become a popular choice due to its high SERS effect, good biocompatibility, and tuneable optical properties [19,20,21]. Recently, various graphene–metal nanocomposites have been developed as SERS substrates and have shown superior application value. In Liang’s work, plasmonic and chemical enhancements were explored by comparing different graphene-based Au systems, such as CVD-G/Au, rGO/Au, and GO/Au; meanwhile, these systems were able to detect biomolecules of adenine with a low detection limit [22]. In Ghosh’s work, directed microwave-assisted self-assembly of Au nanoparticle dimers was used to build Au–Graphene–Au dimer SERS substrates [23]. In Raghavan’s work, the single molecule detection of dopamine and serotonin was achieved by graphene–Au nanopyramid heterostructure platforms [24]. In Stefan’s work, a novel method was reported for the label-free detection of cyanotoxin molecules based on a direct assay utilizing a graphene-modified surface plasmon resonance (SPR) aptasensor [25]. From these reports, it can be seen that the graphene–Au composite structure had an excellent performance in many properties during the Raman testing process, such as uniformity, stability, and biocompatibility. However, only a few graphene–Au SERS substrates can effectively combine all these advantages, which is mainly due to the fact that the performance of the graphene–Au composite structure is often constrained by the morphology and properties of the substrate. Usually, the achievement of corresponding and advantageous performance needs a specific structure, and it is difficult to merge several characteristics [26,27,28].

Here, the graphene film was prepared using the chemical vapor deposition (CVD) method, and a layer of Au nanoparticles (AuNPs) was deposited on the surface by magnetron sputtering to obtain the SERS substrate. Large-area high-quality CVD graphene exhibits extremely high mechanical strength, which ensures that the graphene–Au film can be transferred intactly to different substrates for constructing SERS substrates and will not damage its advantages. In this work, G/Au films were transferred to different substrates to obtain SERS substrates. Due to adjustable SERS enhancement effects induced from chemical and plasmonic enhancement, the G/Au film substrate showed an excellent SERS effect, and the limit of detection (LOD) for R6G was 10^−5^ M. Moreover, the intensity of the Raman signal collected at different positions on the substrate showed high uniform, which proved the reliability of the signal. After transferring the G/Au film to different materials, their SERS effect became different, and additional detection functions could be designed and realized by selecting a suitable target substrate. In order to compare the enhancement effects of different composite SERS substrates, the same batch of G/Au films was transferred to simple silicon wafers, AgNPs/Si substrates, and PDMS films. In the analysis of the results, it was shown that the transfer of the G/Au film to the s AgNPs/Si substrate can provide additional enhancement effects. The detection limit of R6G reduced from 10^−9^ M to 10^−11^ M, which proves that this is an effective way to prepare ultra-sensitive SERS substrates. The transparent and flexible SERS substrate constructed by transferring the G/Au film to the PDMS film showed strong practicability, which can measure MG on the shrimp shell with a concentration of 10^−5^ M by in situ detection. Therefore, the G/Au film can be assembled on different structures to achieve different detection functions, and the use of different properties of the substrate can be realized for different practical applications such as soil, fish, and shrimp. The G/Au film has broad application prospects.

## 2. Materials and Method

### 2.1. Materials

Polydimethylsiloxane (PDMS, C_2_H_6_OSi)_n_, Acetone (CH_3_COCH_3_, −AR, 99.5%), alcohol (C_2_H_6_O, 99.7%), high-purity copper foil (Cu, 99.99%), and rhodamine 6 G (C_28_H_31_N_2_O_3_Cl, AR) were purchased from Sinopharm Chemical Reagent Co., Ltd. (Shanghai, China), and malachite green (C_23_H_25_N_2_·C_2_HO_4_·0.5C_2_H_2_O_4_, AR) and Ferric chloride (FeCl_3_, 98%) were purchased from Shanghai Aladdin Biochemical Technology Co., Ltd. (Shanghai, China). All these materials were used without further purification.

### 2.2. Preparation and Transfer Process of G/Au Film

Graphene was grown on high-purity copper foil using the CVD method, and the carbon source gas was methane. By growing it in a hydrogen environment at 1000 degrees Celsius for 30 min, large-area continuous single-layer to double-layer graphene could be obtained. Then, a layer of Au was sputtered directly on the graphene-grown copper foil to obtain the G/Au film. The transfer of the G/Au film used a conventional wet transfer method, in which the above-mentioned copper foil was floated in a solution of iron chloride (FeCl_3_) and until the copper foil was completely etched. After that, a clean glass slide was used to transfer the G/Au film to deionized water to remove residual ferric chloride. After repeated cleaning 3 times, the G/Au film was directly drawn out using the target substrate and dried, and the transfer process was completed.

### 2.3. Raman Detection of R6G

First, 5 μL of R6G was added to the substrate (1 cm × 1 cm) and then rinsed with alcohol. After the substrate was completely dry, the Raman signal of R6G was detected.

### 2.4. SERS Experiments

Raman spectra were collected using a 532 nm incident laser with an intensity of 0.48 mW, and the diameter of the spot was about 1 μm. The diffraction grid and integration time were respectively set as 600 g/mm and 8 s. During the SERS detection, 5 μL of analyte molecule solution was dropped on the substrate surface and dried to complete the sample preparation process.

## 3. Results

### 3.1. Characterization of the G/Au Film

**Figure 1:**
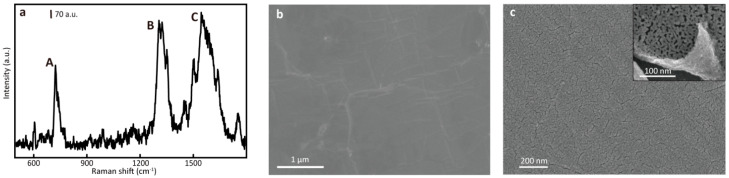
(**a**,**b**) The Raman spectroscopy and SEM images of Cu foil with CVD graphene grown, and (**c**) Cu foil with G/Au film (the insert shows the G/Au film after the transfer process).

The SEM image of the graphene grown by CVD is shown in Figure 1. The surface of the copper foil was observed, and the SEM topography image is shown in Figure 1b. The surface was covered with a transparent graphene film, which covered almost all areas, and the surface was very flat and uniform, which proves the high crystal quality. In order to prove and characterize the growth quality of CVD graphene, the graphene/copper foil was detected by Raman spectra. A total of 15 points were selected at random different positions to collect Raman signals, and the average spectra are shown in Figure 1a. The intensity ratio of the G peak at 1580 cm^−1^ to the 2D peak at 2700 cm^−1^ was close to 1:1, and the shape of the 2D peak was sharp and clear, which proves that the graphene grown on the copper foil was double-layered. In addition, the D peak intensity at 1350 cm^−1^ was quite low, which means that graphene had fewer defects and displayed high growth quality. After depositing the Au layer by magnetron sputtering, the SEM image of the G/Au substrate is shown in Figure 1c. After the Au deposition was completed, an uneven Au film appeared on the surface of the graphene. Then, the copper foil was etched, and the G/Au film was transferred to the silicon wafer, seen from the high-resolution SEM image shown in the Figure 1c insert. The Au film can be regarded as composed of discontinuous gold AuNPs, and there were trenches of nano-gaps between the AuNPs. The diameter of the raised Au nanoparticles and the width of the trenches were less than 100 nm and less than 10 nm, respectively, which is beneficial to the formation of strong plasmon resonance.

### 3.2. Explore the Best SERS Properties of the G/Au Film

**Figure 2:**
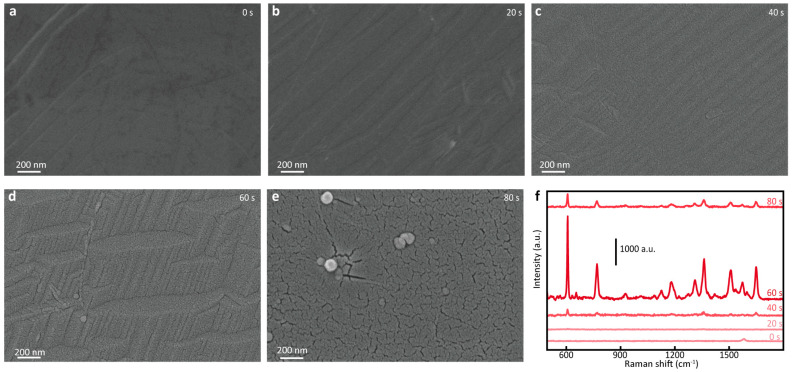
(**a**–**e**) The SEM images of G/Au substrates with Au sputtering times of 0 s, 20 s, 40 s, 60 s, and 80 s, respectively. (**f**) The Raman spectra of R6G with a concentration of 10^−5^ M collected on G/Au substrates with different sputtering times of Au.

For the SERS performance of the prepared G/Au substrate, the main contribution was the plasmon resonance between AuNPs, which can be adjusted by changing the sputtering thickness of the gold layer. The Au sputtering time was set to 20 s, 40 s, 60 s, and 80 s to prepare G/Au substrates with different morphologies, and their SEM images are shown in Figure 2. It can be concluded from the results (shown in Figure 2b–e) that, accompanied by the sputtering time becoming longer, on the one hand, the clusters and gullies of AuNPs became obvious, which means that the distribution of hot spots changed. On the other hand, the Au layer gradually became flat and continuous macroscopically, and large agglomerates began to appear after 80 s of sputtering, which also affected the properties of the G/Au substrate. In order to obtain the best SERS performance, the R6G solution with a concentration of 10^−5^ M was selected as the analyte for Raman testing. A total of 5 μL of R6G solution was added dropwise to the surface of the above substrate, and the Raman spectrum measured after drying is shown in Figure 2f. It can be seen from the figure that, at the beginning, as the Au layer gradually thickened, the SERS effect of the substrate gradually increased; after the sputtering time reached 60 s, the Raman enhancement effect rapidly weakened. In conclusion, the G/Au substrate had a significant Raman enhancement effect, and its enhancement ability first increased and then decreased as the thickness of the Au layer increased. This trend of change was in line with our expectations. As the thickness of the Au layer continued to increase, the content of Au increased, and the hot spots formed between the AuNPs became denser and more efficient when the deposition time was 0–60 s. There are two reasons for the increase of hot spot density: (1) with the increase of Au sputtering time, the content of Au on the graphene surface also increases, which leads to the enhancement of the local surface plasmon resonance effect of the surface; (2) with the increase of Au sputtering time, the gap between Au NPs on the graphene surface become smaller, which leads to the enhancement of the local surface plasmon resonance coupling effect between Au NPs. However, when excessive Au accumulation was over 60 s, together, they tended to form a smooth surface, and a large number of nano-gaps were filled, resulting in the loss of the number of hot spots, thereby weakening the SERS effect. In other words, Au nanoparticles formed with an evaporation time close to about 60 s are most conducive to the construction of highly sensitive G/Au SERS substrates.

### 3.3. SERS Performance of the G/Au Film

**Figure 3:**
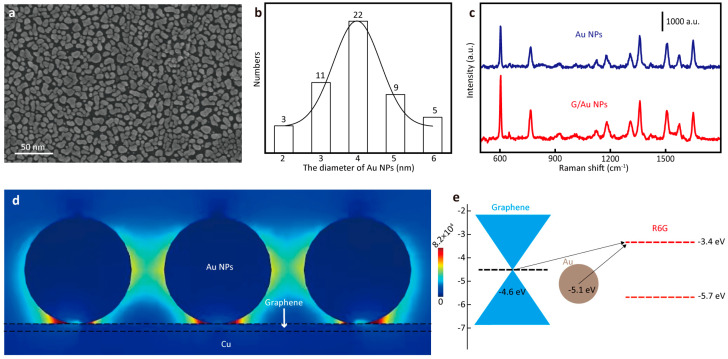
(**a**) The SEM of G/Au substrate; (**b**) the diameter of Au NPs; (**c**) Raman spectra of R6G collected from Au NPs and G/Au NPs; (**d**) simulated electric field distributions in different structures, and the cross section is at the y-z plane; (**e**) the charge transition channel.

In order to further measure the SERS properties of the G/Au substrate, a set of experiments was designed and implemented. Firstly, since the morphology of Au particles is a key factor affecting Raman properties, SEM images of gold on graphene films were taken (Figure 3a), and it can be seen that Au appeared as an irregular spherical shape on the graphene film. Secondly, in order to explore the contribution of gold nanospheres and graphene in the G/Au substrate to SERS enhancement, the SERS properties of Au NPs and G/Au NPs were measured, respectively. As shown in Figure 3c, we collected Raman signals of R6G (10^−5^ M) on Au NPs and G/Au NPs, respectively, and we found that G/Au NPs had better SERS properties than Au NPs. To further quantify the enhanced capabilities of G/Au NPs, we used a generally accepted formula to determine the SERS enhancement factors (EF) of substrates, which is shown below:

(1) $$EF_{\mathrm{AE}}=\frac{I_{\mathrm{SERS}}}{I_{\mathrm{RS}}}\times \frac{N_{\mathrm{RS}}}{N_{\mathrm{SERS}}}\times \frac{P_{\mathrm{RS}}}{P_{\mathrm{SERS}}}$$

where I_SERS_ and I_RS_ represent, respectively, the Raman signal intensities of SERS spectra and the original spectra obtained on pure substrate. The value of N_RS_/N_SERS_ can be considered to be approximately equal to the concentration ratio of the analyte solution [29,30], and P_SERS_ and P_RS_ refer to laser power used by SERS and normal Raman scattering intensity, respectively. In our experiments, different concentrations of the same volume of analyte were dropped on the equal areas of the different substrates, and thus, the N_RS_/N_SERS_ can be estimated as:

(2) $$\frac{N_{\mathrm{RS}}}{N_{\mathrm{SERS}}}=\frac{C_{\mathrm{RS}}\times V_{\mathrm{RS}}\times N_{A}}{C_{\mathrm{SERS}}\times V_{\mathrm{SERS}}\times N_{A}}=\frac{C_{\mathrm{RS}}\times V_{\mathrm{RS}}}{C_{\mathrm{SERS}}\times V_{\mathrm{SERS}}}$$

where N_A_ is Avogadro’s constant, C (C_RS_ and C_SERS_) is analyte concentration, and V (V_RS_ and V_SERS_) is the corresponding volume under the laser spot. In this work, V_RS_ and the V_SERS_ were almost equal. Thus, EF_AE_ can be further simplified as follows:

(3) $$EF_{\mathrm{AE}}=\frac{I_{\mathrm{SERS}}}{I_{\mathrm{RS}}}\times \frac{C_{\mathrm{RS}}}{C_{\mathrm{SERS}}}\times \frac{P_{\mathrm{RS}}}{P_{\mathrm{SERS}}}$$

Data were obtained from the spectrum and brought into the formula, and then the estimated EF could be calculated as 1.40 × 10^5^.

We further simulated the electric field distribution G/Au structures. In order to obtain accurate simulation results, SEM images of Au captured in Figure 3a were analyzed for particle size. Figure 3b shows the diameter of Au NPs. Figure 3c shows the simulated electric field distributions of Au NPs. We found that the hotspots were mainly distributed among Au NPs, which is related to the resonance coupling effect of plasmon [2], and the EF of the simulated electric field was 8.2 × 10^4^. We found that the experimental and theoretical values of EF were different; thus, we hypothesized that there was a CM of graphene during Raman detection. To better understand the chemical enhancement, we analyzed the charge transition between the substrate and the molecule. Figure 3e shows the charge transition channel. The Fermi level of Au was −5.1 eV. The Fermi level of graphene was −4.6 eV. The LUMO and HOMO levels of R6G were −3.4 eV and −5.7 eV, respectively. Under the irradiation of a 532 nm laser, electron transitioned from the Fermi level of graphene to the LUMO level of R6G. At the same time, another charge transition channel was the Fermi level of gold to the LUMO level of R6G. During the charge transition process, the energy difference was smaller than the photon energy provided by the 532 nm laser, achieving a chemical enhancement effect.

**Figure 4:**
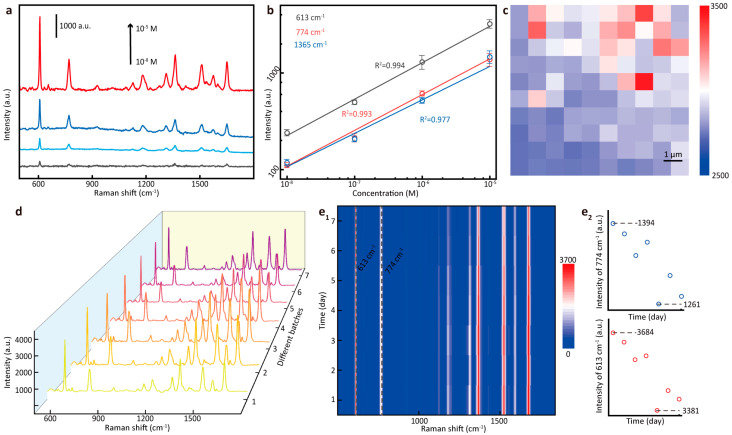
(**a**) Raman spectra of R6G with different concentrations (10^−5^ M to 10^−8^ M) on G/Au substrate. (**b**) Peak intensity alteration over different concentrations of R6G under logarithmic coordinates. (**c**) Color mapping for the Raman spectra of the peak intensities at 613 cm^−1^ (10^−8^ M R6G) measured on 100 random points. (**d**) Raman spectra of R6G with different batches (1–7) of G/Au substrates. (**e**) Raman spectra of R6G collected from G/Au substrate within 7 days.

We made a comprehensive assessment of the SERS property of G/Au substrate. First, in order to characterize the detection sensitivity of the substrate, R6G solutions of different concentrations were dropped on the same substrate as the analyte and Raman spectra were collected, and they are shown in Figure 4a. It can be seen that, even when the concentration of R6G was 10^−8^ M, its characteristic peaks at 613 cm^−1^, 774 cm^−1^, 1365 cm^−1^, etc., could still be clearly displayed, which proves that the substrate has high detection sensitivity. The intensities of characteristic peaks at 613 cm^−1^, 774 cm^−1^, and 1365 cm^−1^ in the spectra were selected, respectively, for linearity analysis, and the result is shown in Figure 4b. It can be proven that the intensities of the same characteristic peaks exhibited good linearity in the Raman spectra of R6G solutions of different concentrations, which can prove that the Raman signal was collected on the substrate with high reliability. To evaluate the Raman enhanced uniformity of substrate, the SERS mapping of the peak intensities at 613 cm^−1^ was measured in an area of 10 × 10 μm^2^ on the G/Au substrate. The step-size of the spectra collection was set to 1 μm, which means that the SERS mapping consisted of spectra collected by 100 points. As shown in Figure 4c, the color distribution in the mapping was uniform, indicating that the signal intensity of the spectra only fluctuated in a small range. To evaluate the repeatability of the substrate, the Raman properties of seven different batches of substrate were tested. As shown in Figure 4d, the Raman properties of different batches of substrates remained stable, indicating that the substrate had high repeatability. Usually, nano Au is an ideal electromagnetic enhancement unit for SERS sensing. Due to its stable chemical properties, it is not easy to oxidize when stored in the air for a long time, and SERS performance was stable. As shown in Figure 4e, the Raman properties of the substrate could remain stable for seven days. In summary, the G/Au substrate has excellent SERS performance and has application potential.

### 3.4. The Feasibility of the G/Au Film Transfer

**Figure 5:**
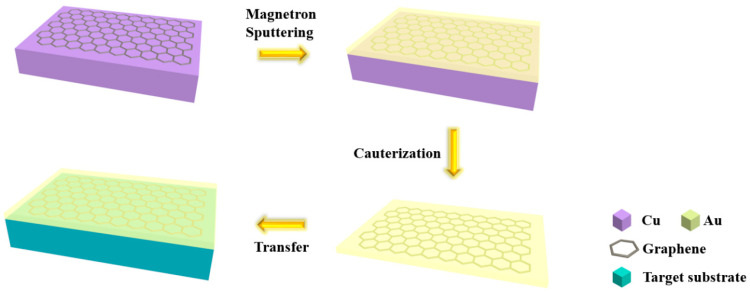
The transfer process diagram of the G/Au film substrate.

In addition, the G/Au film could also be separated from the copper foil and transferred to other substrates, and the process diagram is described in Figure 5. The Cu foil was floated with G/Au film on the surface of a FeCl_3_ solution, and we waited until it was completely etched to obtain a clean G/Au film. Then, the G/Au film could be directly drawn out using the target substrate to make the two combine. In order to verify the quality of the graphene after the transfer, wafer was used as the target substrate, and the measured SEM image of the substrate is shown in Figure 1c. Comparing the images after and before transfer, it can be found that, although the G/Au film cracked to a certain extent after transfer, it was still a large-scale uniform film, which proves the feasibility of substrate transfer.

### 3.5. SERS Performance of the Au/G/Ag NWs

**Figure 6:**
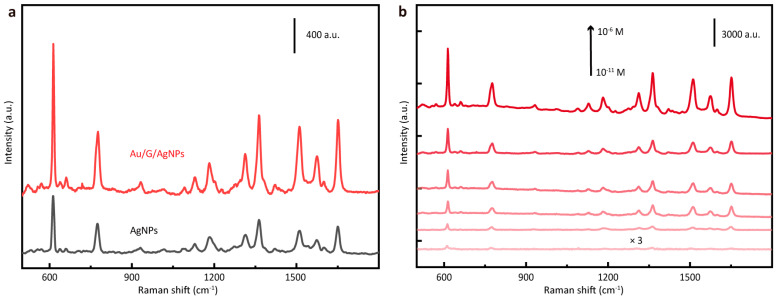
(**a**) Comparison of Raman spectra before and after transferring G/Au film to AgNP substrates (R6G 10^−7^ M). (**b**) Raman spectra of R6G with different concentrations (10^−6^ M to 10^−11^ M) on G/Au substrate.

Through this transfer method and by selecting a suitable target substrate, the advantages of the G/Au film could be fully utilized. Moreover, a thin film tens of nanometers thick does not significantly affect the topography of the substrate and can provide additional Raman enhancement effects. A layer of Ag nanoparticles was dropped on the silicon wafer as the SERS substrate. Then, the G/Au film was assembled on the surface of the substrate, and the changes in its SERS performance were compared. The R6G solution with a concentration of 10^−7^ M was selected as the analyte, and the Raman spectra of pure AgNPs substrate and substrate covered with the G/Au film were collected and are shown in Figure 6a. From the comparison between the two, it can be seen that, after the assembly of the G/Au film, the intensity of each characteristic peak in the spectrum was significantly enhanced, which proves that the G/Au film can be assembled with other SERS substrates into a composite substrate and improve detection sensitivity. In order to quantify the enhancement effect of the substrate more intuitively, different concentrations of R6G solution were used for testing to explore the limit of detection. It can be seen from the Raman spectra (shown in Figure 6b) that the limit of detection of the substrate for R6G was 10^−11^ M, and the estimated EF was calculated to be about 6.80 × 10^7^, which is stronger by several magnitude orders than the detection ability of the simple G/Au film substrate.

**Figure 7:**
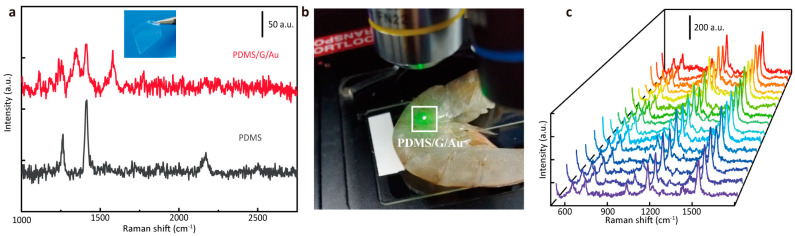
(**a**) The Raman spectra measured on the PDMS film after the transfer and the pure PDMS film. (**b**) The in situ detection process of MG on the surface of shrimp. (**c**) Raman spectra collected at 15 points randomly selected on the substrate.

In addition, the G/Au layer can also be transferred to a flexible substrate to achieve detection tasks that are difficult for rigid substrates. To prove the feasibility of this solution, the G/Au layer was transferred to a PDMS film to prepare a flexible and transparent SERS substrate (shown in Figure 7a insert). The Raman spectra measured on the PDMS film after the transfer and the pure PDMS film are shown in the inset of Figure 7a. It can be seen that the characteristic peaks of graphene could be measured on the transferred substrate, which proves that the transfer was complete. In situ inspection can be directly performed using the substrate without sample processing, and it is suitable for food safety and environmental inspection. Shrimps were soaked in MG solution with a concentration of 10^−4^ M for one night and then rinsed twice. The PDMS substrate and the aforementioned AgNP substrates were used for detection. The PDMS substrate could be directly attached to the surface of the shrimp for detection (shown in Figure 7b), while the rigid AgNP substrate could only be dropped on the washing liquid of the rinse of shrimp on the surface of the substrate. From the measured Raman spectra, the characteristic peaks of MG could be clearly detected on the PDMS substrate. In order to prove the reliability of the signals collected by the flexible PDMS substrate, 15 points were randomly selected on the substrate attached to the surface of the shrimp to collect Raman spectra, and they are displayed in Figure 7c. It can be seen that each Raman spectrum contained the characteristic peaks of MG, and the peak intensities were relatively uniform, which proves that the flexible PDMS substrate can perform effective in situ detection without damaging the sample.

## 4. Conclusions

Here, we constructed a G/Au film using a simple method, and it could be transferred to different substrates to form various composite SERS substrates. The G/Au structure can make the EM and CM work together and achieve a higher enhancement effect. For testing with R6G solution, the estimated EF can reach ca. 1.40 × 10^5^. In addition, the G/Au film, with high mechanical strength, large specific surface area, and good biocompatibility, is beneficial for Raman detection. Transferring it to AgNP substrates and PDMS flexible films can further improve sensitivity and in situ detection functions, respectively, which proves the feasibility of the transferable G/Au film SERS substrate.

## Data Availability

The original contributions presented in the study were included in the article. Further inquiries can be directed to the corresponding authors.
